# The role of Sox9 in mouse mammary gland development and maintenance of mammary stem and luminal progenitor cells

**DOI:** 10.1186/s12861-014-0047-4

**Published:** 2014-12-20

**Authors:** Gautam K Malhotra, Xiangshan Zhao, Emily Edwards, Janel L Kopp, Mayumi Naramura, Maike Sander, Hamid Band, Vimla Band

**Affiliations:** Department of Genetics, Cell Biology and Anatomy, College of Medicine, University of Nebraska Medical Center, 985805 Nebraska Medical Center, Omaha, NE 68198-5805 USA; Departments of Pediatrics and Cellular and Molecular Medicine, University of California-San Diego, La Jolla, CA 92093-0695 USA; Eppley Institute for Research in Cancer and Allied Diseases, and Fred & Pamela Buffett Cancer Center, University of Nebraska Medical Center, 985950 Nebraska Medical Center, Omaha, NE 68198-5950 USA; Departments of Biochemistry & Molecular Biology, College of Medicine, University of Nebraska Medical Center, 985805 Nebraska Medical Center, Omaha, NE 68198-5805 USA; Departments of Pathology & Microbiology and Pharmacology and Neuroscience, College of Medicine, University of Nebraska Medical Center, 985805 Nebraska Medical Center, Omaha, NE 68198-5805 USA; Departments of Pharmacology and Neuroscience, College of Medicine, University of Nebraska Medical Center, 985805 Nebraska Medical Center, Omaha, NE 68198-5805 USA

**Keywords:** SOX9, Stem cells, Luminal progenitor cells, Mammary gland development, Cre-lox, Knockout

## Abstract

**Background:**

Identification and characterization of molecular controls that regulate mammary stem and progenitor cell homeostasis are critical to our understanding of normal mammary gland development and its pathology.

**Results:**

We demonstrate that conditional knockout of Sox9 in the mouse mammary gland results in impaired postnatal development. In short-term lineage tracing in the postnatal mouse mammary gland using Sox9-CreER driven reporters, Sox9 marked primarily the luminal progenitors and bipotent stem/progenitor cells within the basal mammary epithelial compartment. In contrast, long-term lineage tracing studies demonstrate that Sox9+ precursors gave rise to both luminal and myoepithelial cell lineages. Finally, fate mapping of Sox9 deleted cells demonstrates that Sox9 is essential for luminal, but not myoepithelial, lineage commitment and proliferation.

**Conclusions:**

These studies identify Sox9 as a key regulator of mammary gland development and stem/progenitor maintenance.

**Electronic supplementary material:**

The online version of this article (doi:10.1186/s12861-014-0047-4) contains supplementary material, which is available to authorized users.

## Background

Development and maintenance of adult tissues and organs is orchestrated by a pool of resident stem/progenitor cells which are required to meet the needs of tissue remodeling and regeneration associated with physiological and pathological states. Understanding the molecular basis of stem/progenitor maintenance during mammary gland development is of substantial interest given the physiologically critical role of the organ, the dynamic nature of the organ with dramatic changes during sexual and reproductive cycles, and the extremely high prevalence of cancer of mammary gland [1-3]. Mammary transplant studies have established the existence of multipotent mammary stem cells (MaSCs) in the adult mammary gland, with a remarkable capacity to regenerate a functional organ [4-6]. More recently, fluorescence-activated cell sorting (FACS) analyses, based on cell surface markers (e.g., Lin^-^/CD24^+^/CD29^hi^; CD49f^hi^), has allowed for enrichment of MaSCs with mammary gland regenerative potential [7,8]. Transplant of a single MaSC isolated using these approaches can generate a functional mammary gland comprised of both major mammary epithelial cell lineages: luminal and myoepithelial cells [7,8]. Recent reports, however, have suggested caution in assigning MaSC function based on mammary gland regenerative potential in transplantation assays as it may not fully correlate with MaSC function in situ [9-11]. These studies suggest the need to verify the transplant-based conclusions using more definitive *in situ* lineage tracing studies.

Studies using gene knockout or transgenic expression, lineage tracing and regenerative models have led to the identification of a number of molecular pathways that control mammary development by impinging on MaSCs and/or progenitor cell populations. Such studies have demonstrated the role of Notch, Wnt, and LGR5 in mammary gland developmental decisions [9,12,13].

We have previously described the *in vitro* propagation of immortalized human mammary epithelial stem/progenitor cell lines that can be induced to differentiate along the luminal or myoepithelial pathway depending on media conditions [14-16]. An RNA expression screen of parental cells vs. their myoepithelial progeny identified a number of genes whose expression was restricted to bipotent parental cells. Here, we focus on one of these candidate genes, Sox9 (sex-determining region Y [SRY]-box 9 protein) which is a high mobility group box transcription factor that has been demonstrated to play critical roles during embryogenesis and in the development, differentiation, and lineage commitment of a number of organ system [17]. Genetic studies have implicated Sox9 in the maintenance of stem or progenitor cells in the hair follicle, liver, pancreas, and intestine [18-23]. These findings, together with our human MaSC vs. myoepithelial cell expression profiling [14], suggest that Sox9 may physiologically regulate mammary gland development and mammary stem/progenitor cell function. Indeed, in a recent study ectopic expression of Sox9 together with Slug was shown to be sufficient in reprograming mature luminal mammary epithelial cells into MaSCs while Sox9 expression by itself converted these cells into luminal progenitors [24].

Collectively, the findings presented above are consistent with a physiological role of Sox9 in mammary development and MaSC homeostasis. However, this has not been directly tested. Here, we describe studies using mammary gland-directed conditional knockout (cKO) of Sox9 together with Sox9-Cre-mediated activation of reporters for lineage tracing to directly establish a novel role of Sox9 in mammary gland development and maintenance of mammary stem and luminal progenitor cells.

## Results

### Conditional Sox9 deletion results in defective mammary gland development

We have previously characterized immortal human mammary epithelial lines that indefinitely maintain stem/progenitor cell characteristics *in vitro* and these can be induced to differentiate into luminal or myoepithelial progeny [14-16]. Whole genome RNA expression differences between parental cells and their myoepithelial progeny identified Sox9 as one of the transcription factors enriched in undifferentiated parental cells (Additional file 1: Figure S1A). Knockdown of Sox9 using shRNA showed its requirement for the proliferation of these stem/progenitor cell lines (Additional file 1: Figure S1B, C). To further explore the role of Sox9 in a mouse model, we isolated mouse mammary epithelial cells from Sox9^fl/fl^ mice and induced the complete deletion of Sox9 *in vitro* by infecting these cells with an adenovirus expressing Cre-GFP or only GFP as a control) (Additional file 1: Figure S2A). In keeping with human mammary stem/progenitor cell line results, *in vitro* deletion of Sox9 in mouse mammary epithelial cells resulted in a profound inhibition of proliferation as compared to control cells (Additional file 1: Figure S2B).

To examine the physiological consequence of Sox9 deletion in the mammary gland, we crossed Sox9^fl/fl^ mice [25] with mouse mammary tumor virus (MMTV)- Cre mice, which have been established to promote gene deletion in the epithelial compartments of the mammary gland [26]. MMTV-Cre; Sox9^fl/fl^ pups allow for mammary gland specific deletion, allowing an analysis of the impact of Sox9 deletion on mammary gland development.

**Figure 1:**
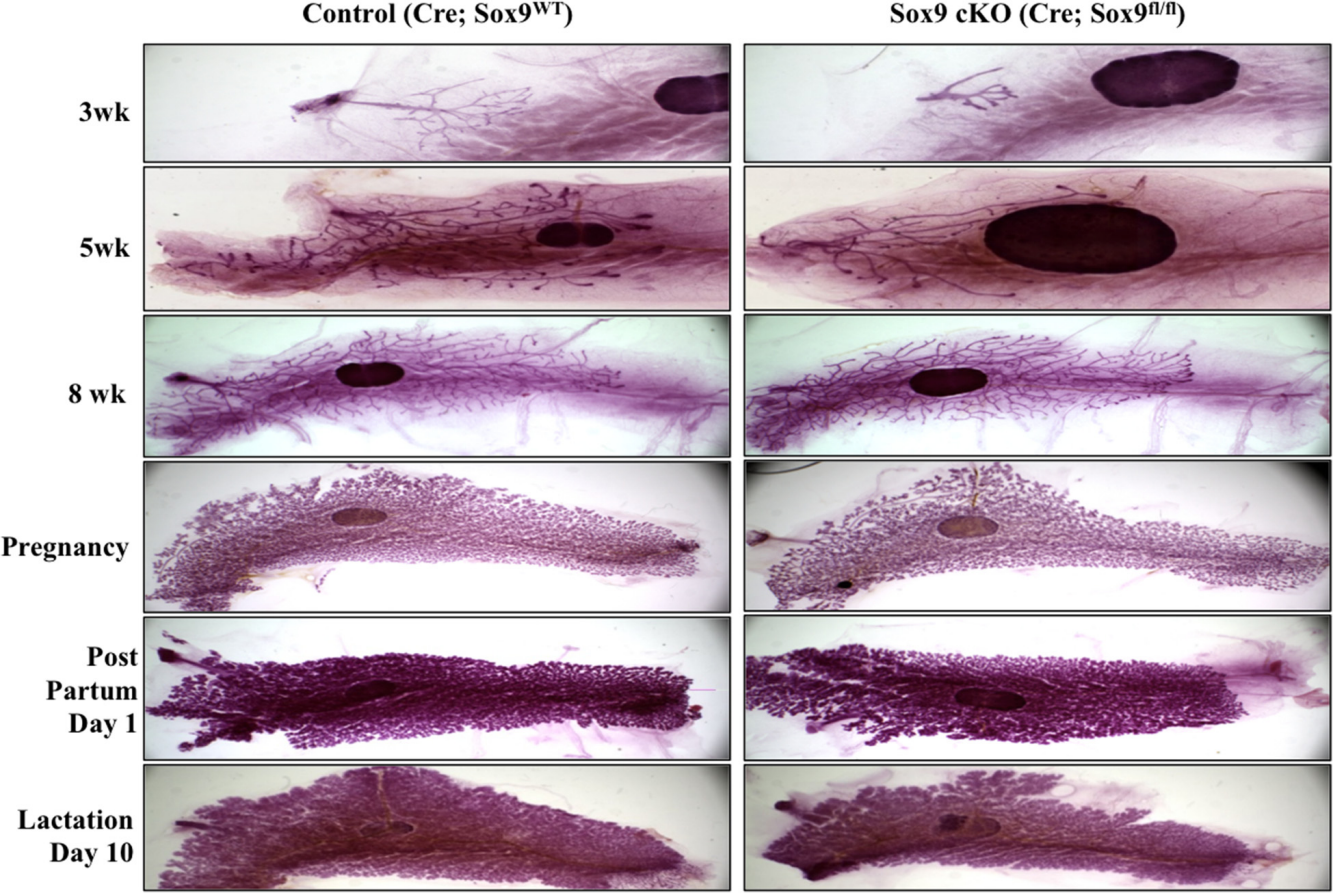
**Deletion of Sox9 results in mammary gland defects in 3 and 5 weeks old mice.** Whole mount analysis of mammary glands from control (MMTV-Cre; Sox9^wt/wt^) and Sox9 cKO (MMTV-Cre; Sox9^fl/fl^) mice. Mammary glands were harvested at 3, 5, 8 weeks, mid-pregnancy, lactation day 1, and lactation day 10 and stained with carmine alum and analyzed under the microscope.

The mouse mammary gland undergoes substantial developmental changes during early postnatal life [2]. Comparison of whole mounts of mouse mammary glands derived from three-week old Sox9 cKO mice revealed clear developmental defects (Figure 1). Compared to control mammary glands, the cKO mammary gland growth was stunted with substantially less ductal outgrowths and side branching. The cKO mammary gland development continued to lag at 5 weeks of age, with considerably less secondary ductal branching. Notably, however, cKO mammary glands at 8 weeks (and beyond; not shown) displayed minimal or no defects in ductal branching or the extent of overall growth (Figure 1). Furthermore, the increased branching and alveologenesis associated with pregnancy and lactation in Sox9 cKO was comparable to that of control mice (Figure 1). Thus, the Sox9 cKO mice show a delay in the development of the mammary gland with a pronounced early phenotype followed by an apparent recovery.

### Sox9 is essential for mouse mammary stem and luminal progenitor cell function

**Figure 2:**
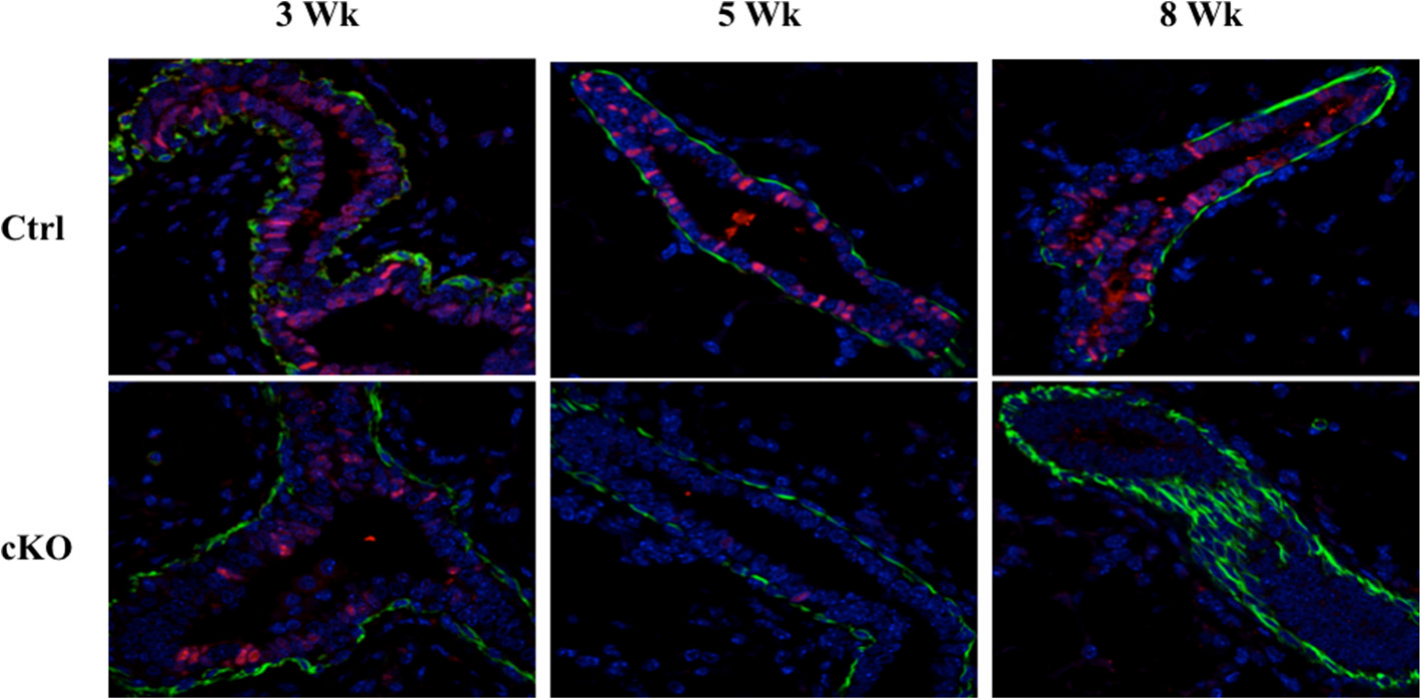
**Sox9 deleted cells are lost over time.** Immunostaining of control (MMTV-Cre; Sox9^wt/wt^) and Sox9 cKO (MMTV-Cre; Sox9^fl/fl^) mouse mammary glands cut sections (3, 5, 8 weeks old mice) with Cre antibody (red). Green and blue represent anti-SMA and DAPI staining, respectively.

The gradual recovery of the developmental phenotype seen in Sox9 cKO mammary glands beyond 8 weeks of age is suggestive of either a transient role of Sox9, or an inability of Sox9-null stem/progenitors to contribute to progeny and compensation by stem/progenitors that did not undergo Cre-mediated Sox9 deletion due to mosaic MMTV-Cre mediated gene deletion. We therefore analyzed the expression of MMTV-Cre in our bi-transgenic mice. The mammary glands of control mice (MMTV-Cre; Sox9^wt/wt^) showed that Cre expression is indeed mosaic, with expression in ~50% of epithelial cells throughout development (Figure 2). Importantly, the proportion of Cre-expressing epithelial cells in the Sox9 cKO mammary glands was markedly diminished (Figure 2). At 3 weeks, when the developmental delay is most pronounced, there is a small percentage of Cre expressing cells. However, the proportion of Cre- expressing mammary epithelial cells is further diminished by 5 weeks, and we were unable to find any such cells in 8-week old Sox9 cKO mice, while such cells were abundant in the age matched control mice (Figure 2). The gradual loss of Cre-expressing mammary epithelial cells in cKO mice supports the conclusion that Sox9 expression is critical for mammary gland development and that Sox9 deleted cells are incapable of contributing to further mammary gland development.

Unfortunately, direct staining of Sox9 was not possible due to non-specific staining within the mammary gland. We tested several anti-Sox9 antibodies (Millipore Cat: AB5535; LSBio Cat: LS-B5761/29528; Abnova, Cat: PAB12736; AbCam Cat: ab3697, ab71762, Ab59252) but were unable to detect specific Sox9 staining in luminal cells. The antibodies tested showed either no reactivity in western blotting or IHC staining, or non-specific reactivity within the myoepithelial cells (that do not express Sox9 mRNA) (Additional file 1: Figure S13A). The most commonly used antibody in the literature (Millipore) [23,24,27] also showed non-specific reactivity with myoepithelial cells in both western blotting and immunofluorescence assays (Additional file 1: Figure S3A, B).

### Lineage tracing reveals that Sox9-expressing cells are precursors of both luminal and basal mammary epithelial compartments, and suggests a role of Sox9 in the maintenance of luminal progenitor pool

Sox9 has been established to play key developmentally regulated roles in the maintenance of stem or progenitor compartments as well as lineage differentiation in many tissues and organs [17-23]. While analyses using ectopic Sox9 expression or knockdown are consistent with a similar functional role of Sox9 in mammary gland development, experimental evidence for any such roles *in vivo* is currently lacking. Our findings that mammary gland development is delayed in MMTV-Cre driven Sox9 cKO mice (Figure 1) strongly suggested a potential role of Sox9 function in MaSCs and/or lineage-restricted progenitors. To experimentally assess this, we carried out lineage-tracing analyses to determine if Sox9 expressing cells give rise to mature mammary lineages. For this purpose, we crossed the Sox9-CreER^T2^ mice, which express a tamoxifen-inducible CreER^T2^ fusion under the control of Sox9 promoter (hence Cre is only expressed in Sox9+ cells after induction with tamoxifen) [28] with either CAG-eGFP or ROSA26-LacZ reporter mice [29].

**Figure 3:**
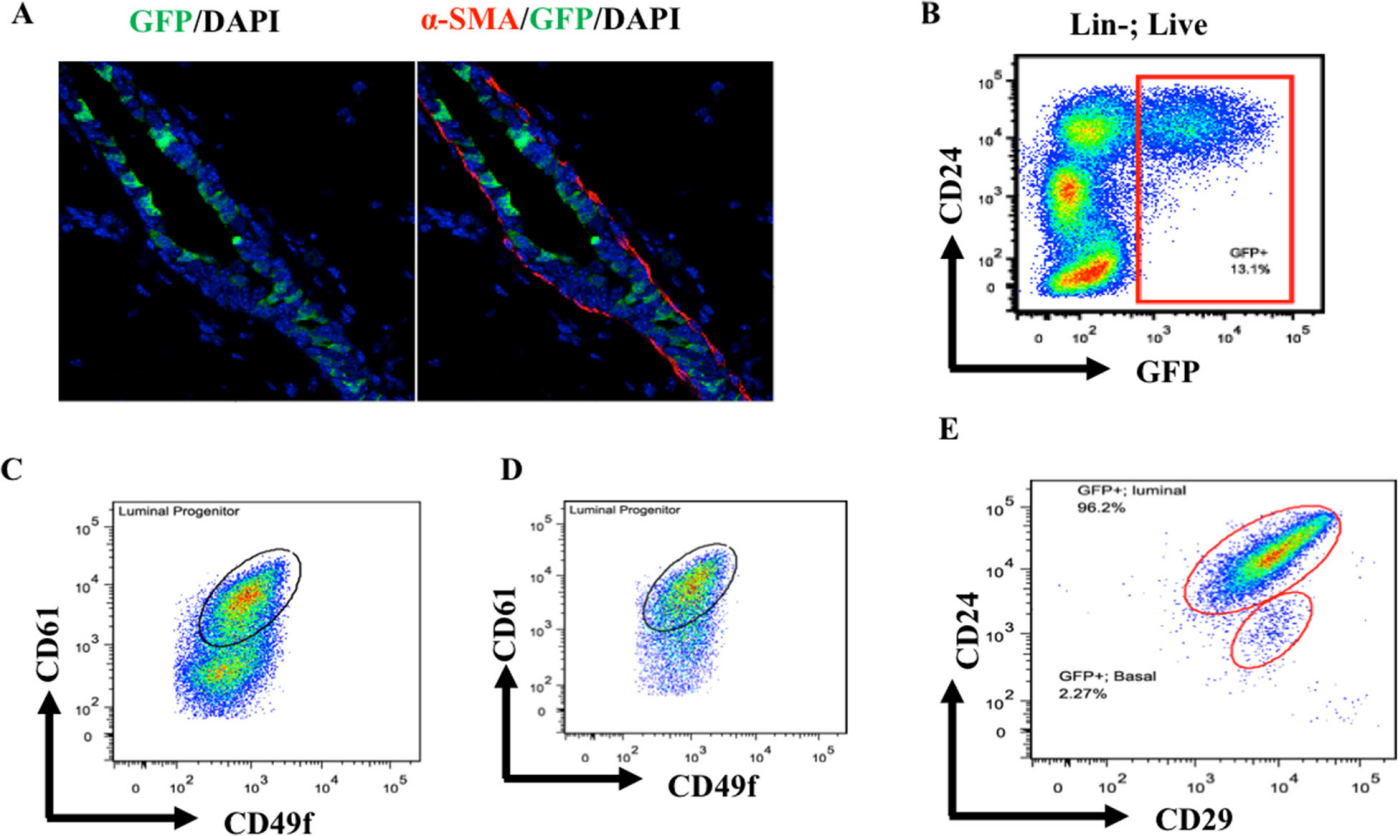
**Short-term linage tracing shows that Sox9 is exclusively expressed in luminal cells. (A)**. Sox9-CreER^T2^; CAG-EGFP mice were injected with tamoxifen. 72 hr after injection, the mice were sacrificed for mammary gland staining and FACS analysis. Sections of the mouse mammary gland were stained with GFP (green) and myoepithelial cell marker (α-SMA) (red) antibodies and DAPI (staining nuclei, blue). **(B)** Flow cytometry analysis of CD24 expression of GFP-positive and -negative cells isolated from above tamoxifen treated mouse mammary glands. **(C)**. Flow cytometry analysis of all CD24+ luminal cells (both GFP+ and GFP-) isolated from the above tamoxifen treated mouse mammary glands using CD61 and CD49f expression. This demonstrates that luminal cells of the mammary gland are comprised of CD61+ luminal progenitors and CD61- differentiated luminal cells. **(D)**. CD61, CD49f flow cytometry analysis of only GFP^+^ cells isolated from above tamoxifen treated mouse mammary glands shows that GFP+ cells are mostly CD61+ luminal progenitors. **(E)** CD24, CD29 flow cytometry analysis of GFP positive cells isolated from above tamoxifen treated mouse mammary glands.

Tamoxifen was administered to adult virgin (>8 weeks) Sox9-CreER^T2^; R26R-LacZ or Sox9-CreER^T2^; CAG-eGFP mice and mammary glands were analyzed after 72 hours. At 72 hours post-induction, analysis of tissue sections for GFP+ cells was seen almost exclusively in the luminal compartment (Figure 3A). Flow cytometry analyses are concordant with this finding, as the vast majority of GFP^+^ cells (shown as red panel) resided within the CD24^hi^ luminal compartment (Figure 3B). Notably, GFP^+^ population represents only a fraction of the luminal compartment (Figure 3B), suggesting that the Sox9+ cell progeny seen at this time may correspond to an intrinsic subpopulation within the luminal compartment. We hypothesized that this intrinsic subpopulation may represent luminal progenitors within the mammary gland.

To further assess the nature of the Sox9^+^ cells within the luminal compartment, we examined the co-expression of CD61, a marker of luminal progenitors, on the CD24^hi^ luminal population [3,30]. When analyzing all CD24^hi^ (GFP+ and GFP-) cells, we find that there are distinct CD61^hi^ and CD61^lo^ populations (Figure 3C). However, when analyzing only the CD24^hi^;GFP+ population (Sox9 expressing luminal cells) we find that the vast majority of GFP^+^ cells reside within the CD61^hi^ luminal progenitor subpopulation (Figure 3D). These results support the conclusion that Sox9 is expressed within the luminal progenitor population. Notably, when analyzing the CD24^hi^;GFP+ population, we did observe a small fraction of GFP^+^ cells within the CD24^lo^/CD29^hi^ compartment (Figure 3E) that is known to include bipotent MaSCs [7,9].

**Figure 4:**
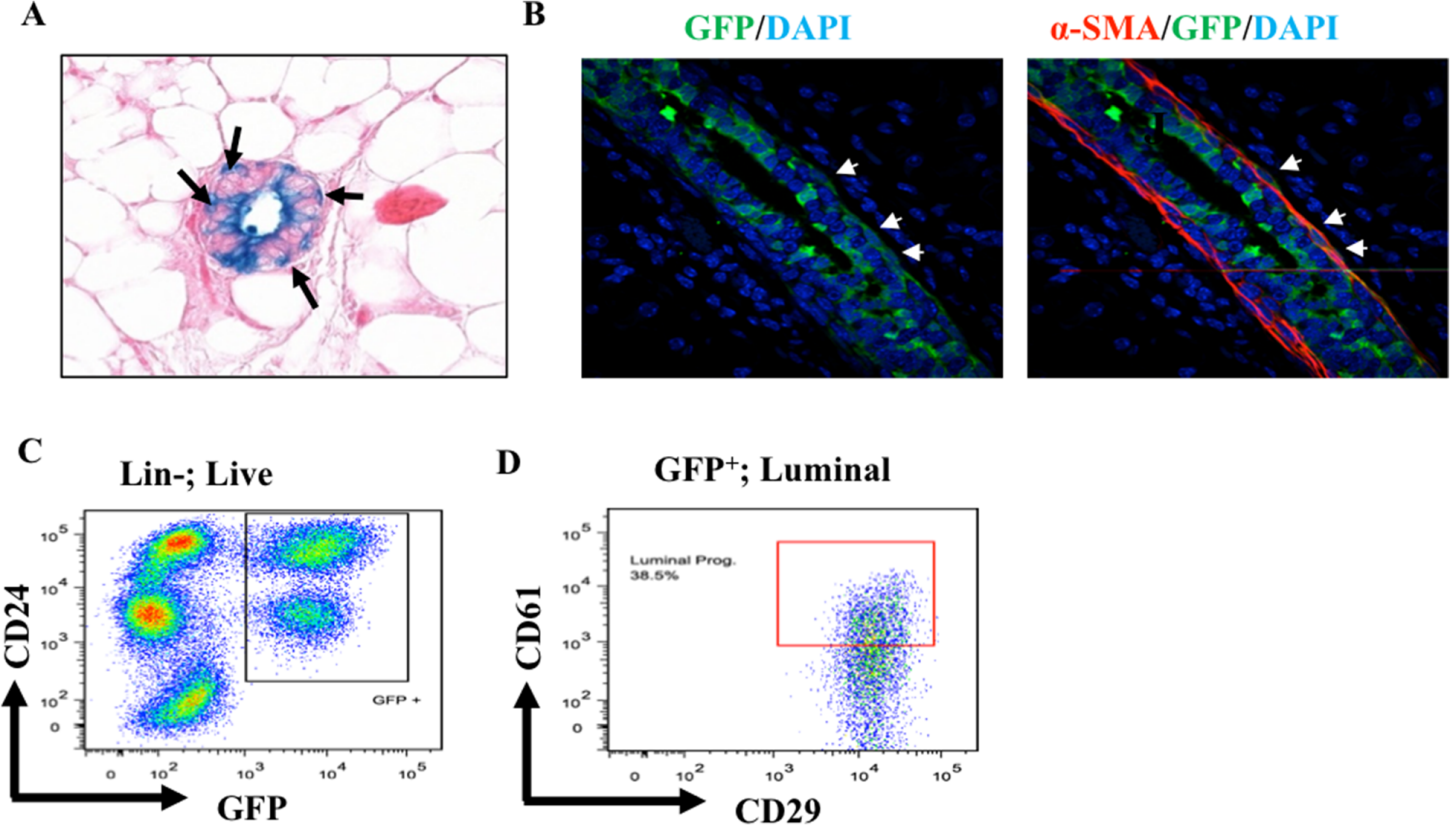
**Long-term linage tracing shows that Sox9 expressing cells are capable of giving rise to both luminal and myoepithelial lineages (A)**. X-gal stained tissue section of adult virgin Sox9-CreERT2; R26R-LacZ mammary gland injected with TM and harvested after 4 weeks. Arrows point to reporter activity in myoepithelial cells. **(B)**. GFP stained tissue section of adult virgin Sox9-CreER^T2^; CAG-EGFP mammary gland injected with TM and harvested after 4 weeks. Arrows highlight the progeny of Sox9 expressing cells within the myoepithelial layer **(C)**. Flow cytometry analysis of CD24, GFP expression of GFP positive and negative cells isolated from above adult virgin mice mammary glands. **(D)**. Flow cytometry analysis of CD61, CD29 expression of GFP^+^ cells isolated from above adult virgin mice mammary glands.

To determine if Sox9 expressing cells represented bipotent MaSCs with an ability to differentiate into multiple cell lineages, we performed long term lineage tracing experiments by administered tamoxifen to these mice and harvesting the mammary glands after 4 weeks. Staining of histological sections showed that after 4 weeks of propagation, Sox9 expressing progeny (seen as LacZ+) made up a proportion of both luminal and myoepithelial layers (Figure 4A). This result was more clearly established by immunofluorescence staining for GFP together with anti-smooth muscle actin (SMA) which helps demarcate the myoepithelial (SMA^+^) and luminal (SMA^-^) layers (Figure 4B). Both SMA^+^GFP^+^ and SMA^-^GFP^+^ cells were observed suggesting a common ancestry. We also prepared single-cell suspensions of harvested mammary glands and subjected these to flow cytometry to analyze the distribution of GFP^+^ cells within the CD24^hi^ (luminal) and CD24^lo^ (basal) mammary epithelial compartments, the latter including myoepithelial cells and MaSCs [31]. A substantial fraction of both basal and luminal compartments was GFP^+^ (Figure 4C), indicating that Sox9-expressing precursors gave rise to both of the major mammary epithelial compartments. Dual staining for CD29 and CD61 showed that GFP+ progeny included both CD61^hi^ luminal progenitors and CD61^lo^ differentiated luminal cells (Figure 4D).

**Figure 5:**
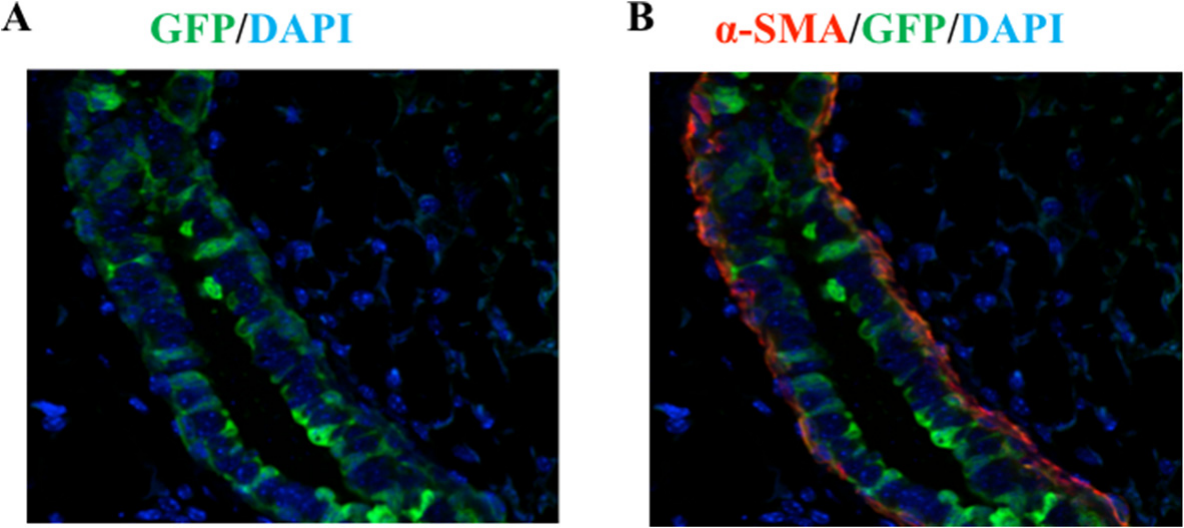
**Non-inducible long-term linage tracing shows that Sox9 is expressed in luminal and myoepithelial cells.** A non-inducible Sox9-Cre knock-in mouse model was used to assess the contribution of Sox9+ cells to mature lineages. Sox9-Cre mice were crossed with CAG-EGFP mice. Sox9-Cre; CAG-EGFP mammary gland cut sections were stained with anti-GFP (green) and α-SMA (red) antibodies. DAPI staining is blue. **(A)** GFP staining shows Sox9 expressing cells contribute to both luminal and myoepithelial lineages. **(B)** α-SMA (red) is used to highlight the myoepithelial cells.

Finally, we also performed an independent long-term lineage-trace using a non-inducible Sox9-Cre knock-in mouse model [32] to assess the contribution of Sox9+ cells to mature lineages. For this purpose, we generated Sox9-Cre; CAG-eGFP bi-transgenic mice [32,33]. Analysis of whole mount mammary glands demonstrated uniform GFP expression throughout the mammary ductal system in adult virgin (>8 weeks) Sox9-Cre; CAG-eGFP mice (Figure 5A). Immunofluorescence analyses of mammary gland sections stained for GFP and myoepithelial marker SMA revealed uniform GFP expression in both luminal and myoepithelial layers (Figure 5B). Collectively, our lineage-tracing results demonstrate that Sox9-expressing cells function as precursors of all mature lineages as well as luminal progenitors, consistent with the functional role of Sox9 in bipotent MaSCs and luminal progenitors.

### Lineage-tracing analyses of Sox9 cKO mice show loss of Sox9-null progeny in the luminal but not in the basal/myoepithelial compartment

**Figure 6:**
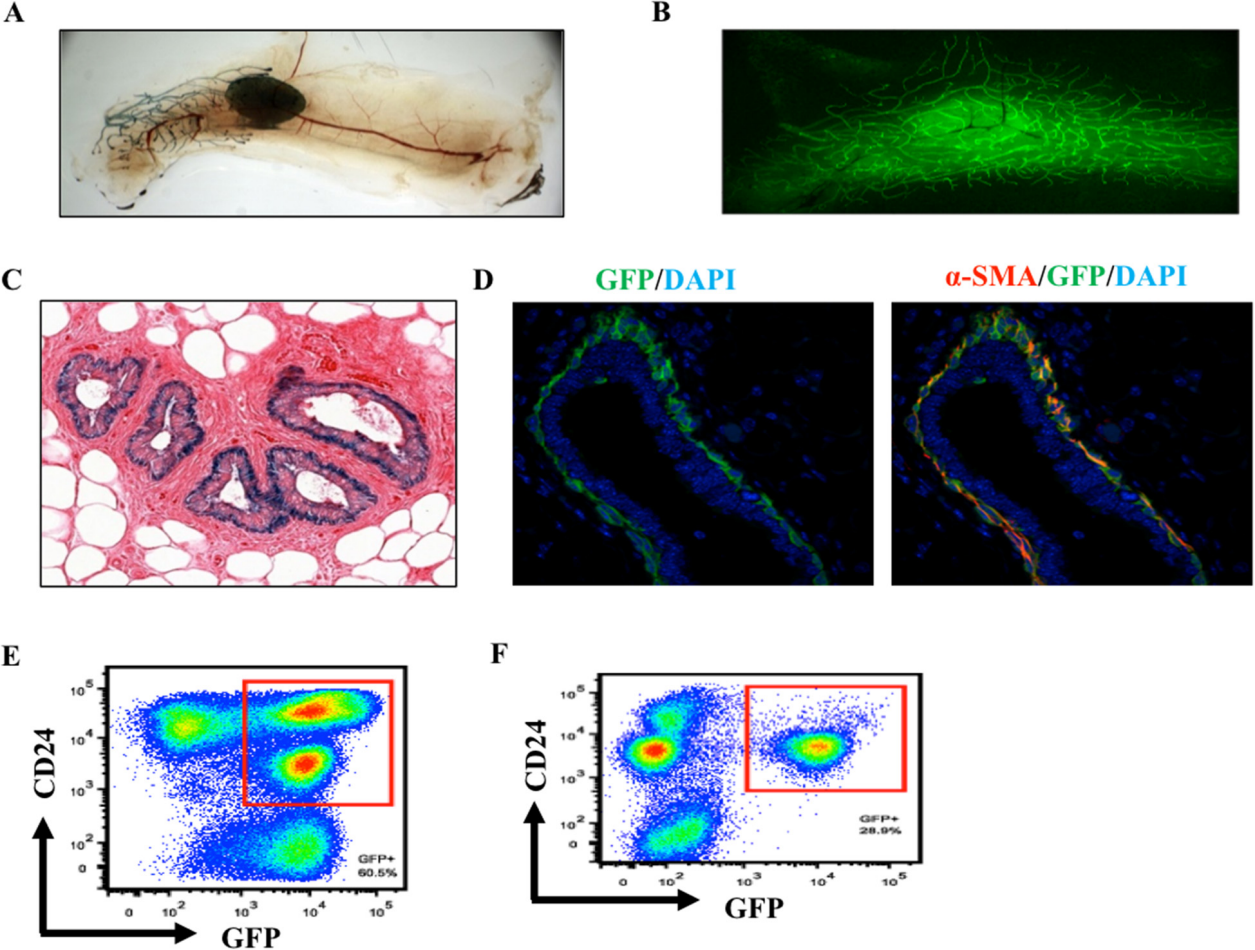
**Sox9 deleted luminal cells are lost over time. (A)**. LacZ staining of whole mount mammary glands from 5 week old Sox9 cKO (R26R; MMTV-Cre; Sox9^fl/fl^) mouse. **(B)**. GFP in whole mount 8 week old Sox9 cKO (CAG-EGFP; MMTV-Cre; Sox9^fl/fl^) was visualized with fluorescence microscope. **(C)**. Section of mammary glands from 5-week old Sox9 cKO (R26R; MMTV-Cre; Sox9^fl/fl^) mouse, stained with X-gal. **(D)**. Section of mammary glands from 8 week old Sox9 cKO (CAG-EGFP; MMTV-Cre; Sox9^fl/fl^) mouse, immuno-labeling with anti-GFP (green) and anti-α-SMA (red) antibodies. **(E)**. CD24, GFP FACS analysis of cells isolated from control (CAG-EGFP;MMTV-Cre; Sox9^wt/wt^) mouse mammary glands. **(F)**. CD24, GFP Flow cytometry analysis of cells isolated from Sox9 deletion (MMTV-Cre; Sox9^fl/fl^; CAG-EGFP) mouse mammary glands.

To determine the fate of mammary epithelial precursors in which Sox9 was deleted using MMTV-Cre, we used further crosses to incorporate the CAG-eGFP and ROSA-LacZ reporters into MMTV-Cre; Sox9^fl/fl^ strain. As we observed depletion of Cre-expressing cells in both luminal and myoepithelial compartments of 8-week old MMTV-Cre-driven Sox9 conditional KO mice (Figure 2), we anticipated the lineage-trace to show an absence of Sox9-null progeny in both mammary cell compartments. However, whole mount analysis showed uniform LacZ and GFP staining throughout the mammary glands of 5 and 8 week old mice (Figure 6A-B). Notably, LacZ or GFP staining of histological sections revealed that the progeny of Sox9 deleted precursors was restricted to the basal/myoepithelial layer with essentially complete absence from the luminal compartment (Figure 6C-D). FACS analyses demonstrated that GFP^+^ progeny was present in both luminal and basal compartments in the control (MMTV-Cre; Sox9^wt/wt^; CAG-eGFP) mice (Figure 6E). In contrast, the mammary glands of mice with MMTV-driven Sox9 deletion showed a near complete absence of GFP^+^ (sox9-deleted) progeny in the luminal compartment (Figure 6F), consistent with staining results (Figure 6C-D). These results suggest that myoepithelial progeny of Sox9-deleted precursors are viable while their luminal progeny is not.

## Discussion

Sox9 has been established as a key regulator of tissue stem and progenitor cells during development in several organ systems [18-22,28], more recently has been linked to cancer stem cell function [24]. A potential role of Sox9 in regulating mammary stem/progenitor cell functions has emerged from recent studies using ectopic expression and analyses of breast cancer cell lines. However, a physiological role of Sox9 in mammary gland development and mammary stem/progenitor cells remains unknown. Here, we use a conditional gene deletion approach to provide in situ evidence for a physiological role of Sox9 as an important regulator of mammary gland development. We also carried out short and long term lineage-tracing analyses to implicate a role for Sox9 in the maintenance of mammary stem cells and luminal progenitors.

Our conclusion that Sox9 is an essential component of the mammary gland developmental program is based on a substantial delay in post-natal mammary gland ductal elongation and branching in mice with MMTV-Cre directed Sox9 deletion (Figure 1). The defects in development are particularly severe at early time points (3 weeks postnatal) but are followed by gradual recovery, with essential no defects beyond 8 weeks of age. We hypothesized that recovery from the developmental block imposed by Sox9 deletion is overcome by the expansion of mammary epithelial cells in which Sox9 deletion did not occur, due to the known mosaic expression of MMTV-Cre that we used (Figure 2). Unfortunately, direct Sox9 staining was not possible due to lack of specific anti-Sox9 antibodies. Several antibodies that we tested showed either no or non-specific reactivity in western blotting, IHC, IF (Additional file 1: Figure S3). Consequently, we used Cre expression as a surrogate for Sox9 deletion and found that Sox9 deleted cells are lost in cKO mice by 8 weeks (Figure 2). Accordingly, no subsequent defects in mammary gland development were observed during pregnancy and lactation. These results suggest that Sox9 deletion puts the precursors of mammary epithelium at a developmental disadvantage. Our efforts at conditional Sox9 deletion in the mammary gland using a K14-Cre, which is active during embryonic mammary placode stage [34], were not feasible due to perinatal lethality of the successfully targeted mice. However, the absence of mammary gland in a rare short-term survivor confirmed conclusions reached based on the use of MMTV-Cre driven Sox9 knockout mice (data not shown). Our results are reminiscent of more severe phenotypes of gene deletion using K14-Cre vs. MMTV-Cre, reported for GATA-3 deletion [30]. The inducible model presented here provides a tractable system to further examine the cellular and molecular basis of Sox9 function in the mammary gland.

In view of the importance of Sox9 in mammary gland development revealed by our conditional knockout studies, we considered the possibility that Sox9 is important for the function of mammary stem/progenitor populations as these are the sources of developmental growth and expansion of the mammary gland. To test this possibility in an unbiased manner, we carried out lineage-tracing studies using a Sox9-directed Cre to turn on reporters that allowed us to track the progeny of Sox9-expressing precursor populations. When such studies were carried throughout embryonic development (using a non-inducible Sox9-Cre), all mammary epithelial cells, including the luminal and myoepithelial/basal cell layers, were uniformly positive for reporter expression (Figure 5). In keeping with this result, a four week chase following induction of a tamoxifen-inducible Sox9-CreER^T2^ driver in postnatal mice led to the appearance of the progeny of Sox9-expressing precursors in both luminal and myoepithelial/basal cell populations of the mammary gland (Figure 4). These studies suggest that Sox9 expression marks an early bi-potent MaSCs, however, we cannot preclude the possibility that Sox9 may be expressed in both luminal and myoepithelial restricted progenitors. In view of the defects in mammary development upon Sox9 deletion (discussion above), we suggest that Sox9 is an important regulator of the developmental program of MaSCs. Furthermore, we have previously published a report of isolating and immortalizing a human MaSC line that is able to differentiate into the myoepithelial lineage [35]. Western blot data confirms that Sox9 expression is markedly higher in immortal human MaSC lines vs. their progeny differentiated along the myoepithelial lineage (Additional file 1: Figure S2). Furthermore, Sox9 deletion results in inhibition of proliferation in in both human mammary epithelial and mouse mammary epithelial cells (Additional file 1: Figure S1 and S2).

Several recent studies support our conclusion that Sox9 provides a critical function in maintaining MaSCs. For example, ectopic co-expression of Sox9 and Slug reprogramed mature luminal mammary epithelial cells into MaSCs capable of generating mammary gland-like growths upon transplant [24]. Down-regulation of Wnt-1-induced signaling protein-2 (WISP2) in relatively well-differentiated MCF7 breast cancer cell line promoted a cancer stem-like cell phenotype with upregulation of SOX9 [36]. In another study, expression of miR-140, which targeted Sox9 directly, was seen to be lower in breast cancer patients and cancer stem cells and was associated with increased SOX9 expression [37]. High Sox9 expression in basal-like breast cancer correlates with poor prognosis and activation of Wnt/β-catenin pathway, and SOX9 silencing reduced cell proliferation and invasion [38]. Furthermore, overexpression in cell lines increased Wnt signaling transgenic overexpression in murine mammary glands increased secondary ductal branching [38]. As Sox9 is a Wnt target in other stem cell systems [39], and accumulating evidence suggest a key role of Wnt/β-Catenin pathway in the maintenance of MaSCs [9,40], it is reasonable to postulate that Sox9 is an important component of MaSC maintenance by Wnt/β-Catenin and potentially other pathways.

Notably, two significant findings in our study imply a more important role of Sox9 in the regulation of luminal progenitors. First, short term lineage-tracing almost exclusively marked the luminal mammary epithelial layer, and FACS analyses using CD61 confirmed the marked population to be luminal progenitors (Figure 3). Second, when we carried out lineage tracing in the context of Sox9 deletion induced by MMTV-Cre, we noted that progeny of Sox9-deleted precursors was absent from the luminal epithelial compartment but such cells were still present within the myoepithelial/basal compartment and may represent the progeny of restricted myoepithelial progenitors that underwent Sox9 deletion (Figure 6). These results suggest that Sox9 function may be particularly critical for the development of the luminal lineage. Selective labeling of luminal progenitors upon short-term tracing is consistent with this conclusion. This idea is further supported by recent findings that ectopic Sox9 expression in mature mammary epithelial cells converts these into cells expressing markers of luminal progenitors [24].

Taken together, the findings presented in this paper demonstrate a physiological role of Sox9 in mammary gland development. Our results, considered in light of recent publications, implicate Sox9 as a key regulator of the maintenance of mammary stem cells and luminal progenitors. With emerging evidence that Sox9 may contribute to maintenance of breast cancer stem cells, the inducible Sox9 deletion model and the insights presented here should facilitate future efforts to validate Sox9 as a biomarker or therapeutic target in breast cancer.

## Conclusion

In summary, in this study, we show that Sox9 expression is restricted to luminal progenitors and bipotent basal stem/progenitor cells in the postnatal mouse mammary gland. Long-term lineage tracing studies demonstrated that Sox9+ precursors give rise to both luminal and myoepithelial cell lineages. Conditional knockout of Sox9 in the mouse mammary gland results in impaired postnatal development. In vitro knockout SOX9 impaired mouse mammary epithelial proliferation. Indicate Sox9 is an essential regulator of mouse mammary stem/progenitor cells.

## Methods

### Mice

All animal protocols were reviewed and approved by the Institutional Animal Care and Use Committee at the University of Nebraska Medical Center. Sox9-IRES-Cre knock-in mice were a generous gift from Dr. Elaine Fuchs, Rochester, NY. Sox9-CreER^T2^ mice were generated in the Sanders laboratory as described [28]. For conditional deletion, Cre recombinase was expressed under the control of the mouse mammary tumor virus (MMTV) (Line A and D) promoter [26]. Sox9^fl/fl^ and Rosa26^fl-STOP-fl-β-geo^ (R26R) mice were obtained from the Jackson Laboratory (013106 and 003474 respectively). CAG-CAT-eGFP reporter transgenic mice have been previously described [33].

Floxed mice were bred with MMTV-Cre (Line A and D) [26] to generate heterozygous Cre; Sox9^fl/+^ mice; which were then bred with Sox9^fl/fl^ mice to obtain Cre; Sox9^fl/fl^, Cre; Sox9^fl/+^; and non-transgenic Sox9^fl/fl^ mice. These mice were then intercrossed with either R26R or CAG-eGFP mice to trace the fate of Sox9 deleted cells.

### In Vivo tamoxifen induction

Tamoxifen (Sigma T5648) was dissolved in corn oil (Sigma C8267) at a concentration of 20 mg/ml. For inducible lineage tracing studies, Sox9-CreER^T2^; R26R or Sox9-CreER^T2^; CAG-eGFP mice were injected intra-peritoneally with tamoxifen at 5 mg per 40 g body weight. Mice were injected 3 times, once every other day unless otherwise noted. A minimum of n = 3 mice were analyzed for each time point. Adult virgin Sox9-creER mice were used for those experiments.

### Histology and whole mount analysis

For histological examination, the 9^th^ inguinal mammary glands were harvested at various developmental stages and fixed in 10% neutral buffered formalin (Sigma HT501128) for 24 hrs. The glands were paraffin embedded, sectioned (4 μm) and stained with either hematoxylin/eosin or antibodies discussed below.

For whole mount analysis, the 4^th^ inguinal mammary glands were harvested at various developmental stages and fixed in Carnoy’s solution for 3-5 hours followed by incubation in acetone overnight. The following day, mammary glands were rehydrated and stained in carmine alum overnight. Finally, the glands were dehydrated, cleared in xylenes or Histo-clear, and mounted with Omnimount (National Diagnostics). For LacZ staining of whole mount mammary glands, the gland was fixed in 2% paraformaldehyde, 0.25% glutaraldehyde, 0.01% NP-40 in PBS for 1-2 hours. The glands were then washed 3 × 30 min. with 2 mM magnesium chloride, 0.01% sodium deoxycholate and 0.2% NP-40 in PBS. Finally, the glands are stained in the above wash solution supplemented with 30 mM potassium ferricyanide, 30 mM potassium ferrocyanide, and 1 mg/ml X-gal overnight at 30°C. After appropriate LacZ staining, the glands are washed, cleared in 2:1 benzyl benzoate:benzyl alcohol (BABB), mounted and imaged. The glands were counterstained with carmine alum and mounted with Omnimount. For eGFP visualization, mammary glands were harvested immediately after the animal was sacrificed, cleared in BABB, and visualized. Following visualization, glands were fixed and stained with carmine alum, as described above.

### Mammary epithelial cell isolation and flow cytometry

Mouse mammary epithelial cell isolation was carried out as described [31]. In short, mice are individually culled and both sets of the second, third, and fourth mammary glands are aseptically removed. The lymph node is removed from the fourth mammary glands, and all glands are transferred to a 50 ml conical tube with Leibowitz L15 medium (L15) supplemented with 10% fetal bovine serum (FBS). The glands are finely chopped (100 μm) using the McIlwain tissue chopper and transferred to a 50 ml conical tube containing 35 ml of serum free L15, 100 mg of collagenase A, and 50 mg of trypsin. The chopped tissue is incubated at 37°C with slight agitation for one hr. After the incubation the mixture is centrifuged at 250 g to remove the fat and isolate organoids and single cells. The mixture is then washed twice with red blood cell lysis buffer. To remove most of the mammary fibroblasts, the mixture is resuspended in DMEM and plated in large T-75 flasks incubated at 37°C for one hour to allow fibroblasts to attach. Finally, after the one hour incubation, the supernatant containing epithelial organoids was plated in DFCI medium [41] to generate bulk cultures. For single cell isolation, the organoids were further processed by enzymatic disassociation using trypsin and DNAse I. The single cells were then strained through a 0.45 μm filter and counted. Cells were then used for flow cytometry analysis or *in vitro* propagation assays. Cells were first stained with biotin label antibodies for CD45, CD31, and Ter119 (all from BD bioscience). Subsequently, the cells were washed and stained with a cocktail of antibodies including Streptavidin-Alexa-350 (Invitrogen), CD24-BV421 (BD), CD29-PE-Cy7 (eBioscience), CD49f-APC (Biolegend), and CD61-PE (BD), followed by analysis on a BD FACS Aria II. For cell propagation assays, cells were cultured in DFCI media and infected with either Adeno-Cre-GFP or Adeno-GFP. GFP expressing cells were isolated by FACS and plated at 1x10^4^ cells per well for proliferation assay. Cells were counted every other day using a coulter counter.

## Additional file

Additional file 1: Figure S1. Sox9 knockdown inhibits proliferation in human mammary epithelial stem/progenitor cells. (A), Sox9 protein expression in human mammary epithelial stem/progenitor ( MaSCs) and myoepithelial progenitor cells (MPCs) using western blotting with anti-Sox9 antibody. (B), MaSCs expressing scrambled (control) or Sox9 shRNA using western blotting with anti-Sox9 antibody. (C), Proliferation curves of MaSCs control or upon Sox9 shRNA knockdown. Experiment was carried out three times. A representative figure from one experiment with Mean + SD of three replicates is shown. **Figure S2.** Sox9 deletion in Sox9fl/fl mammary epithelial cells inhibits proliferation. (A), mammary fibroblasts or mammary epithelial cells from Sox9fl/fl mice were infected with adenovirus GFP (control) or Cre-GFP (Sox9 KO). Sox9 protein expression was analyzed by western blotting using anti-Sox9 antibody. (B), Proliferation over days was assessed in Sox9fl/fl mammary epithelial cells infected with adenovirus Cre (red) and GFP (blue). **Figure S3.** Western blotting and immunostaining with several anti-Sox9 antibodies. (A), Lysates from human mammary stem/progenitor ( stem) and myoepithelial (myo) cells (known to be negative for Sox9 mRNA expression) were examined for Sox9 protein by western blotting. Please note, several non-specific bands were observed in all antibodies tested with the exception of one antibody (ab71762) which did not show any immunoreactivity in western blotting. Antibodies tested include Millipore Cat: AB5535; LSBio Cat: LS-B5761/29528; Abnova, Cat: PAB12736; AbCam Cat: ab3697, ab71762, Ab59252. (B), Formalin fixed paraffin embedded mammary gland cut sections were prepared from MMTV-Cre; Sox9fl/fl; CAG-eGFP mice and were co-stained with α-GFP (green) and α-Sox9 (red) antibodies (Millipore). Myoepithelial cells, which are clearly deleted for Sox9 (as demonstrated by their GFP expression), are also staining positive for Sox9, which is consistent with the non-specific immunoreactivity seen in western blotting. DAPI (blue) was used for nuclear counterstaining.

## Abbreviations

MaSCs: Multipotent mammary stem cells
cKO: Conditional knockout
