# Supplementary material for: The role of Sox9 in mouse mammary gland development and maintenance of mammary stem and luminal progenitor cells
Source: BMC Dev Biol. 2014 Dec 20;14:47. doi: 10.1186/s12861-014-0047-4 (PMC4297388; doi:10.1186/s12861-014-0047-4)

Supplementary Figures Legends

Fig. S1. Sox9 knockdown inhibits proliferation in human mammary epithelial stem/progenitor cells. (A), Sox9 protein expression in human mammary epithelial stem/progenitor (MaSCs) and myoepithelial progenitor cells (MPCs) using western blotting with anti-Sox9 antibody. (B), MaSCs expressing scrambled (control) or Sox9 shRNA using western blotting with anti-Sox9 antibody. (C), Proliferation curves of MaSCs control or upon Sox9 shRNA knockdown. Experiment was carried out three times. A representative figure from one experiment with Mean+ SD of three replicates is shown.

Fig. S2. Sox9 deletion in Sox9$^{fl/fl}$ mammary epithelial cells inhibits proliferation. (A), mammary fibroblasts or mammary epithelial cells from Sox9$^{fl/fl}$ mice were infected with adenovirus GFP (control) or Cre-GFP (Sox9 KO). Sox9 protein expression was analyzed by western blotting using anti-Sox9 antibody. (B), Proliferation over days was assessed in Sox9$^{fl/fl}$ mammary epithelial cells infected with adenovirus Cre (red) and GFP (blue).

Fig. S3. Western blotting and immunostaining with several anti-Sox9 antibodies. (A), Lysates from human mammary stem/progenitor (stem) and myoepithelial (myo) cells (known to be negative for Sox9 mRNA expression) were examined for Sox9 protein by western blotting. Please note, several non-specific bands were observed in all antibodies tested with the exception of one antibody (ab71762) which did not show any immunoreactivity in western blotting. Antibodies tested include Millipore Cat: AB5535; LSBio Cat: LS-B5761/29528; Abnova, Cat: PAB12736; AbCam Cat: ab3697, ab71762, Ab59252. (B), Formalin fixed paraffin embedded mammary gland cut sections were prepared from MMTV-Cre; Sox9$^{fl/fl}$; CAG-eGFP mice and

were co-stained with α-GFP (green) and α-Sox9 (red) antibodies (Millipore). Myoepithelial cells, which are clearly deleted for Sox9 (as demonstrated by their GFP expression), are also staining positive for Sox9, which is consistent with the non-specific immunoreactivity seen in western blotting. DAPI (blue) was used for nuclear counterstaining.

Fig. S1.

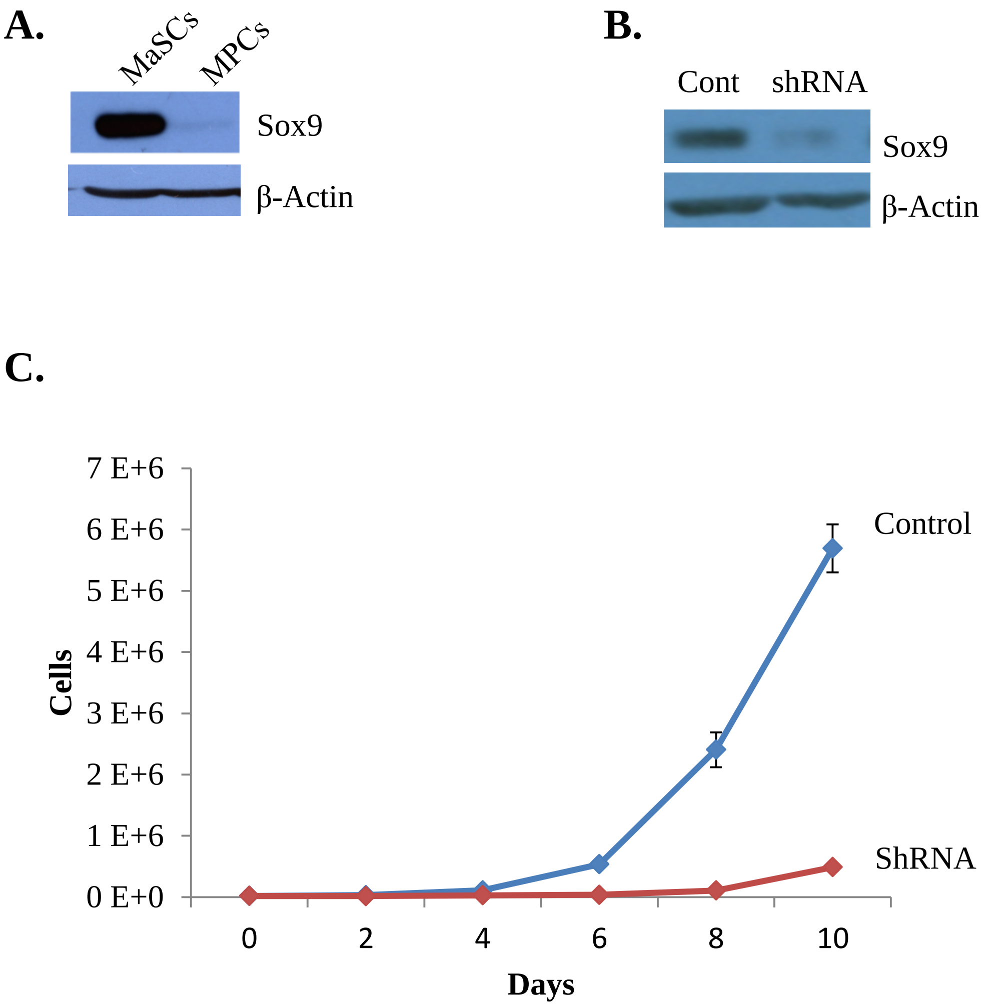
A.
MaSCs
MPCs
Sox9
β-Actin
B.
Cont
shRNA
Sox9
β-Actin
C.
7 E+6
6 E+6
5 E+6
4 E+6
3 E+6
2 E+6
1 E+6
0 E+0
Cells
0
2
4
6
8
10
Days
Control
ShRNA

Fig. S2.

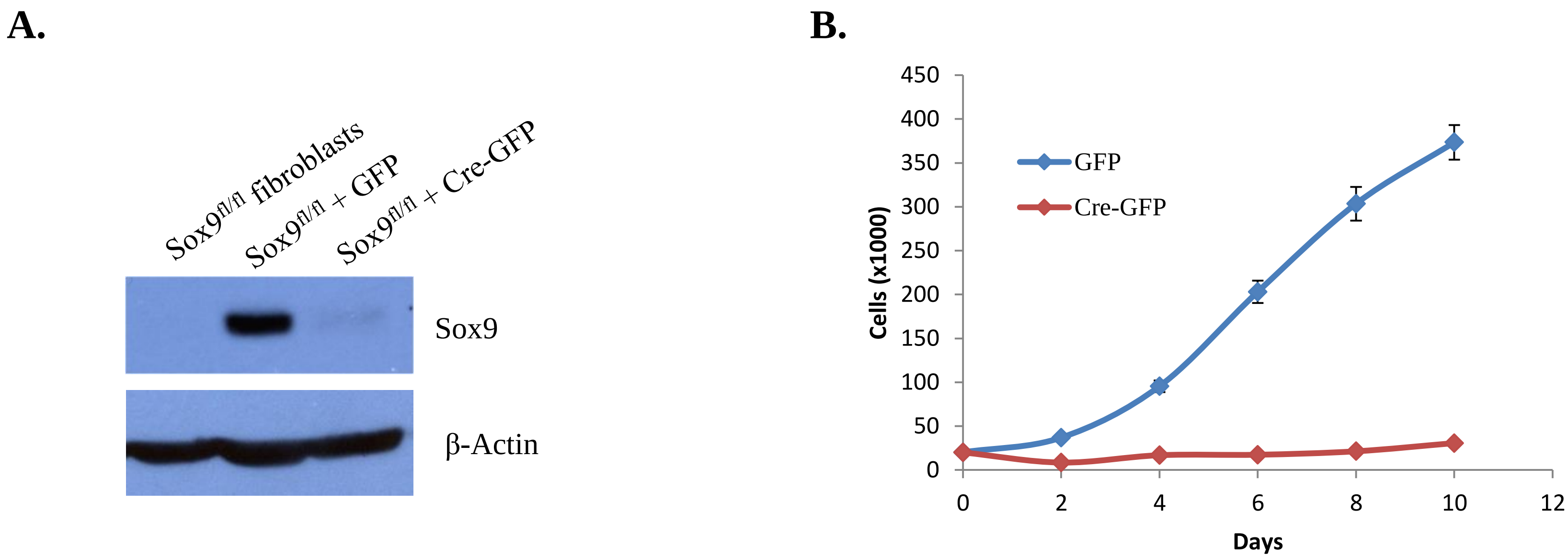
A.
Sox9fl/fl fibroblasts
Sox9fl/fl + GFP
Sox9fl/fl + Cre-GFP
Sox9
β-Actin
B.
GFP
Cre-GFP
Cells (x1000)
450
400
350
300
250
200
150
100
50
0
0
2
4
6
8
10
12
Days

Fig. S3.

A.

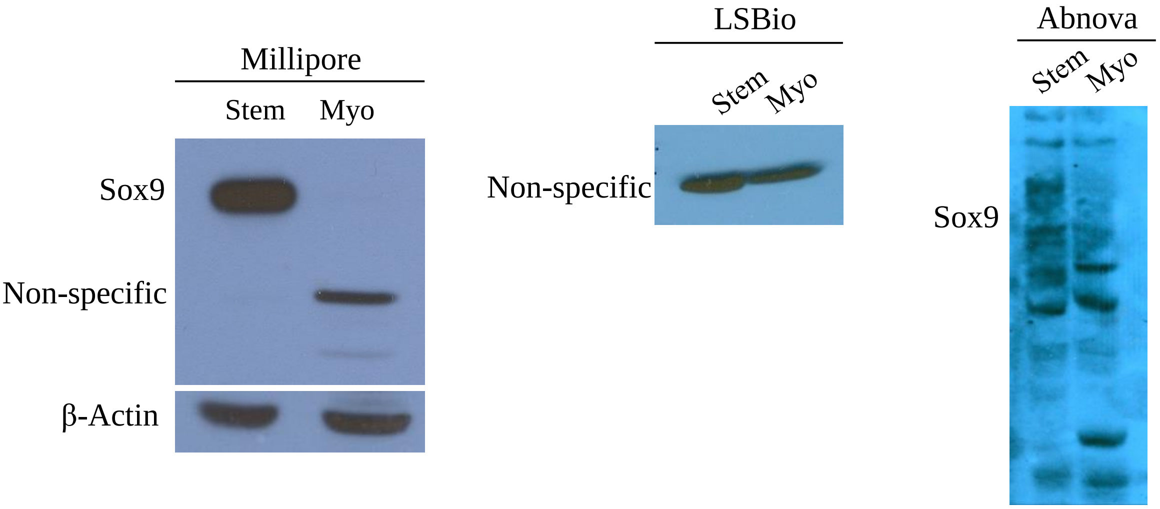
Millipore
Stem
Myo
Sox9
Non-specific
β-Actin
LSBio
Stem
Myo
Non-specific
Abnova
Stem
Myo
Sox9

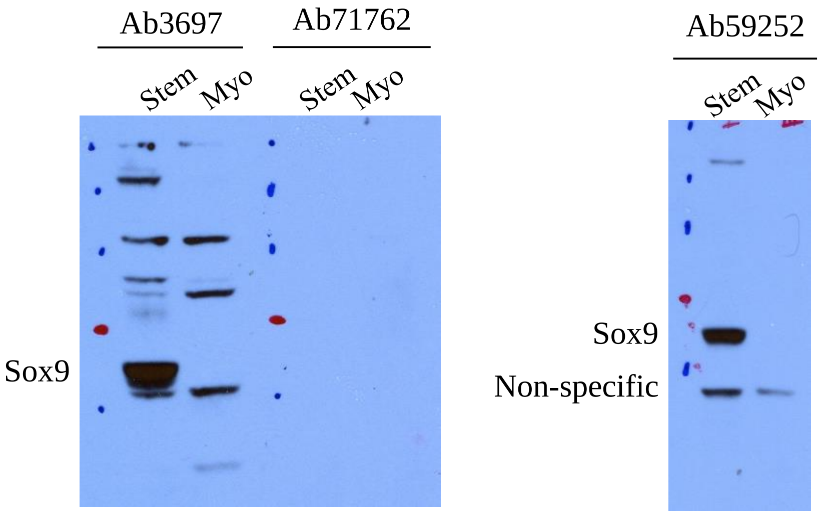
Ab3697
Ab71762
Stem
Myo
Stem
Myo
Sox9
Ab59252
Stem
Myo
Sox9
Non-specific

B.

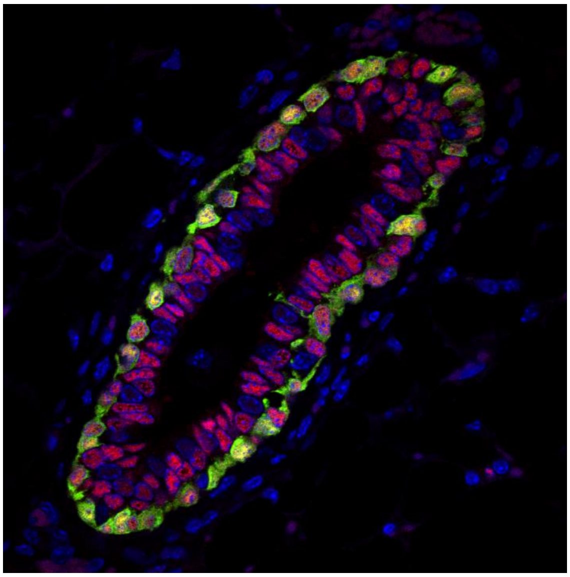

Supplement: Additional file 1: Figure S1. — Sox9 knockdown inhibits proliferation in human mammary epithelial stem/progenitor cells. (A), Sox9 protein expression in human mammary epithelial stem/progenitor ( MaSCs) and myoepithelial progenitor cells (MPCs) using western blotting with anti-Sox9 antibody. (B), MaSCs expressing scrambled (control) or Sox9 shRNA using western blotting with anti-Sox9 antibody. (C), Proliferation curves of MaSCs control or upon Sox9 shRNA knockdown. Experiment was carried out three times. A representative figure from one experiment with Mean + SD of three replicates is shown. Figure S2. Sox9 deletion in Sox9fl/fl mammary epithelial cells inhibits proliferation. (A), mammary fibroblasts or mammary epithelial cells from Sox9fl/fl mice were infected with adenovirus GFP (control) or Cre-GFP (Sox9 KO). Sox9 protein expression was analyzed by western blotting using anti-Sox9 antibody. (B), Proliferation over days was assessed in Sox9fl/fl mammary epithelial cells infected with adenovirus Cre (red) and GFP (blue). Figure S3. Western blotting and immunostaining with several anti-Sox9 antibodies. (A), Lysates from human mammary stem/progenitor ( stem) and myoepithelial (myo) cells (known to be negative for Sox9 mRNA expression) were examined for Sox9 protein by western blotting. Please note, several non-specific bands were observed in all antibodies tested with the exception of one antibody (ab71762) which did not show any immunoreactivity in western blotting. Antibodies tested include Millipore Cat: AB5535; LSBio Cat: LS-B5761/29528; Abnova, Cat: PAB12736; AbCam Cat: ab3697, ab71762, Ab59252. (B), Formalin fixed paraffin embedded mammary gland cut sections were prepared from MMTV-Cre; Sox9fl/fl; CAG-eGFP mice and were co-stained with α-GFP (green) and α-Sox9 (red) antibodies (Millipore). Myoepithelial cells, which are clearly deleted for Sox9 (as demonstrated by their GFP expression), are also staining positive for Sox9, which is consistent with the non-specific immunoreactivity seen in we [file 12861_2014_47_MOESM1_ESM.pdf]
